# Involvement of the FoxO1/MuRF1/Atrogin-1 Signaling Pathway in the Oxidative Stress-Induced Atrophy of Cultured Chronic Obstructive Pulmonary Disease Myotubes

**DOI:** 10.1371/journal.pone.0160092

**Published:** 2016-08-15

**Authors:** Pascal Pomiès, Marine Blaquière, Jonathan Maury, Jacques Mercier, Fares Gouzi, Maurice Hayot

**Affiliations:** 1 PhyMedExp, INSERM U1046, CNRS UMR9214, University of Montpellier, Montpellier, France; 2 Department of Clinical Physiology, CHRU of Montpellier, Montpellier, France; 3 Clinique du Souffle "La Solane", Fontalvie – 5-Santé Group, Osséja, France; University of Louisville School of Medicine, UNITED STATES

## Abstract

Oxidative stress is thought to be one of the most important mechanisms implicated in the muscle wasting of chronic obstructive pulmonary disease (COPD) patients, but its role has never been demonstrated. We therefore assessed the effects of both pro-oxidant and antioxidant treatments on the oxidative stress levels and atrophic signaling pathway of cultured COPD myotubes. Treatment of cultured COPD myotubes with the pro-oxidant molecule H_2_O_2_ resulted in increased ROS production (P = 0.002) and protein carbonylation (P = 0.050), in association with a more pronounced atrophy of the myotubes, as reflected by a reduced diameter (P = 0.003), and the activated expression of atrophic markers MuRF1 and FoxO1 (P = 0.022 and P = 0.030, respectively). Conversely, the antioxidant molecule ascorbic acid induced a reduction in ROS production (P<0.001) and protein carbonylation (P = 0.019), and an increase in the myotube diameter (P<0.001) to a level similar to the diameter of healthy subject myotubes, in association with decreased expression levels of MuRF1, atrogin-1 and FoxO1 (P<0.001, P = 0.002 and P = 0.042, respectively). A significant negative correlation was observed between the variations in myotube diameter and the variations in the expression of MuRF1 after antioxidant treatment (P = 0.047). Moreover, ascorbic acid was able to prevent the H_2_O_2_-induced atrophy of COPD myotubes. Last, the proteasome inhibitor MG132 restored the basal atrophy level of the COPD myotubes and also suppressed the H_2_O_2_-induced myotube atrophy. These findings demonstrate for the first time the involvement of oxidative stress in the atrophy of COPD peripheral muscle cells *in vitro*, *via* the FoxO1/MuRF1/atrogin-1 signaling pathway of the ubiquitin/proteasome system.

## Introduction

COPD is characterized by the progressive development of airflow limitation. The dysfunction and atrophy of skeletal limb muscles are important extrapulmonary manifestations of COPD that also contribute to impaired patient exercise tolerance and reduced survival [1]. Muscle atrophy is generally described as a combination of both increased proteolysis and reduced muscle protein synthesis. In COPD, the expression of markers of the proteolysis pathway, such as the ubiquitin ligases atrogin-1 and MuRF1 and the transcription factors FoxO1 and FoxO3, are increased in the atrophic muscle of patients compared with controls [2–4]. Furthermore, the expression of myostatin, a muscle growth suppressor acting on both the protein synthesis and protein breakdown pathways, is unchanged or increased in atrophied COPD muscle compared with control muscle [3–5]. Nevertheless, some of the results concerning the expression of markers of the protein synthesis pathway in COPD-atrophied muscles compared with controls have been intriguing. Indeed, the expression level of IGF-1 was found to be increased in atrophied COPD muscle [6], while the P-AKT/AKT ratio was unaltered or increased, a process that has been interpreted as an attempt to restore muscle wasting [2,4,6].

Oxidative stress is considered to be one of the most important mechanisms leading to muscle dysfunction and atrophy in COPD patients. For example, exercise-induced oxidative stress, which is reflected by an increase in muscle lipid peroxidation and oxidized proteins, has been implicated in the reduced quadriceps endurance of these patients [7,8]. Furthermore, the correlation between systemic exercise-induced oxidative stress and muscle wasting in COPD patients suggests a causal relation between oxidative stress and muscle atrophy [9]. At a molecular level, H_2_O_2_-induced oxidative stress upregulates expression of atrogin-1 and MuRF1 and induces muscle atrophy, in association with a proteasome-dependent degradation of MHC in C2C12 cells [10–12]. Nevertheless, the involvement of oxidative stress in COPD muscle atrophy has yet to be clearly demonstrated [3].

Using an *in vitro* cellular model, we recently showed that satellite cells derived from COPD patients have normal proliferative and differentiation capacities compared to those of healthy subjects. However, the cultured myotubes from these patients have characteristics of atrophy and elevated oxidative stress similar to those of *in vivo* quadriceps from COPD patients [13]. This *in vitro* model of COPD muscle alteration thus provides a promising basis to explore the signaling pathways involved in the atrophy and elevated oxidative stress of COPD skeletal muscles. Indeed, it provides access to molecular mechanisms that have not been studied thus far or that are very difficult to assess directly in COPD muscle, as such studies would require multiple fresh muscle biopsies from the patients. Therefore, we used this cellular model to investigate whether oxidative stress is involved in the atrophy of COPD skeletal muscle *in vitro*. Oxidative stress levels and atrophic signaling were assessed in cultured COPD myotubes subjected to an H_2_O_2_ pro-oxidant treatment or an ascorbic acid antioxidant treatment.

## Materials and methods

### Study population

Sedentary healthy subjects were defined on the following criteria: age from 57 to 67.5 years, no disease, and less than 150 minutes of moderate-to-vigorous physical activity per week. COPD patients were recruited on the basis of the following criteria: dyspnea and/or chronic cough or sputum production and/or history of exposure to risk factors for the disease, with the diagnosis confirmed by spirometry. The main exclusion criteria were other respiratory diagnosis and exacerbation in the last 2 months. Informed written consent was obtained from all subjects, and the research protocol was approved by the institutional ethics committee of the Montpellier University Hospitals (n°2009-04-BPCO-V2 and n°2011-A00842-39) and conducted in accordance with the Helsinki Declaration and the European guidelines for “good clinical practice.” The pulmonary and muscle function tests were performed at “La Solane” Pulmonary Rehabilitation Center in Osseja (Fontalvie– 5-Santé Group, France), with the usual methods of our group [14].

### Anthropometric data

Weight (W) and height (H) were measured, and the body mass index (BMI) was calculated (BMI = W/H^2^). Fat-free mass (FFM) was estimated using multifrequency bioelectrical impedancemetry (QuadScan 4000, Bodystat, Isle of Man, UK), and the fat-free mass index (FFMI) was calculated (FFMI = FFM/H^2^).

### Pulmonary function tests

All subjects underwent spirometry (Medisoft Body Box 5500, Sorinnes, Belgium). FEV_1_ (forced expiratory volume in 1 second) and FVC (forced vital capacity) were measured, and the FEV_1_/FVC ratio was calculated and compared with normal values [15].

### Exercise testing and peripheral muscle function assessment

A 6-minute walking test was performed in a 30-m corridor. The quadriceps muscle voluntary contraction (qMVC) was assessed with the usual methods of our group [14], and was compared with reference values [16].

### Muscle biopsy procedures

Muscle biopsies were performed in the *vastus lateralis* of the quadriceps using the needle methodology routinely used in our group [17]. One piece of the fresh biopsy was placed in fetal bovine serum (FBS)/10% DMSO in a cryogenic tube, which was progressively frozen to -80°C for 24 hours in a cryobox (Nalgene Mr. Frosty Freezing Container; Thermo Fisher Scientific, Pittsburgh, PA). The cryogenic tube was then placed and conserved in liquid nitrogen until use of the biopsy for myoblast purification.

### Myoblast purification and culture

Small explants of the biopsy were defrosted and placed in a 35-mm collagen Petri dish. Satellite cells were then purified with an anti-CD56 antibody using an immunomagnetic sorting system (Miltenyi Biotec, Bergisch Gladbach, Germany) as previously described in detail [13].

Myoblasts were grown in collagen-coated Petri dishes in DMEM/20% FBS/0.5% Ultroser (proliferation medium). Myoblasts were always cultured at a passage below P4 for the various experiments. When myoblasts reached 80% confluence, myogenic differentiation was induced by changing the proliferation medium for the differentiation medium (DMEM/2% FBS). Myotubes were obtained after 6 days of culture in the differentiation medium.

### Cell treatments

For the pro-oxidant experiment, myotubes cultured in differentiation medium for 5 days were then incubated with 50 μM H_2_O_2_ for 18 hours. These pro-oxidant conditions were chosen because they induce significant oxidative stress-induced cell damage and atrophy, and because they do not induce significant cell death of C2C12 and COPD myotubes, as shown in previous works [12,13]. To study the involvement of the ubiquitin/proteasome pathway, the proteasome inhibitor MG132 was added to the culture medium 1 hour before adding H_2_O_2_. For the antioxidant experiment, myoblasts were incubated with 0.14 mM ascorbic acid 1 day before induction of differentiation. This treatment was then renewed every day until fully differentiated myotubes were obtained, 6 days after induction of differentiation. Treatment of the COPD myotubes with higher concentrations of ascorbic acid induced the detachment of the cells from the culture dishes, indicating that elevated concentrations of ascorbic acid are toxic for cultured cells (data not shown). When myotubes where treated with both H_2_O_2_ and ascorbic acid, COPD cells where incubated with 0.14 mM ascorbic acid as described above, but after 5 days of culture in differentiation medium supplemented with ascorbic acid, 50μM H_2_O_2_ was added to the culture medium for 18 hours.

### Fluorescence microscopy

Myotubes were fixed in PBS/3.7% formaldehyde followed by a 5-min permeabilization in PBS/0.1% Triton X-100. Staining was performed using an anti-troponin T antibody (1/50) and Hoechst 33258. Images were captured at a 5x magnification from at least 5 random fields with an AxioCam MRm CCD camera (Carl Zeiss, Oberkochen, Germany) driven by AxioVision 4 software (Carl Zeiss) on a Zeiss AxioImager M1 microscope (Carl Zeiss). Images were anonymized by giving a random number and the myotube diameter was evaluated in a blinded fashion by 2 investigators: using the ImageJ software (National Institutes of Health, Bethesda, MD), 3 measurements of the diameter were taken along each myotube, for at least 100 myotubes per culture [13].

### SDS-PAGE and Western immunoblotting

Cells were lysed in hypotonic buffer (20 mM Tris pH7.5/10 mM NaCl/1 mM DTT/protease inhibitor cocktail; Sigma-Aldrich, St. Louis, MO), and protein concentration was determined using the Bio-Rad Protein Assay (Bio-Rad Laboratories, Hercules, CA). Proteins were separated by SDS-PAGE and transferred to Immobilon-P PVDF (Millipore, Bedford, MA) membranes. Proteins of interest were revealed by specific antibodies (anti-α-tubulin 1/20,000; anti-phospho-AKT 1/2,000; anti-AKT 1/2,000; anti-MuRF1 1/500; anti-atrogin-1 1/1,000; anti-FoxO1 1/2,000), followed by enhanced chemiluminescence. Scanned radiographs were quantified using the ImageJ software.

### Oxidative stress assessment

ROS production was assessed in myotubes using CellROX Green Reagent (Molecular Probes) as described by the manufacturer. Briefly, 5 μM of the fluorogenic probe was incubated for 45 minutes in differentiation medium at the end of the differentiation process. After 3 washes with PBS, cells were lysed in hypotonic buffer and ROS production was analyzed by fluorometry on an Infinite 200 PRO microplate reader (Tecan Group, Mannedorf, Switzerland).

The OxyBlot Protein Oxidation Detection Kit (Millipore) was used to detect carbonyl group formation into protein side chains (primary antibody at a dilution of 1/150), while HNE-modified proteins were detected with a rabbit polyclonal anti-HNE antibody (1/250; Abcam, Cambridge, UK) for assessment of total lipid peroxidation, as described previously [13].

### Reagents and antibodies

Mouse monoclonal anti-troponin T and anti-α-tubulin were purchased from Sigma-Aldrich, rabbit monoclonal anti-phospho-AKT and rabbit polyclonal anti-AKT were purchased from Cell Signaling Technology (Danvers, MA), and rabbit monoclonal anti-MuRF1, anti-atrogin-1 and anti-FoxO1 were purchased from Abcam. Hoechst 33258, H_2_O_2_ and ascorbic acid were purchased from Sigma-Aldrich, and MG132 from Selleckchem (Houston, TX).

### Quantitative polymerase chain reaction (qPCR) and primers

Total RNA was extracted from myotubes using TRIzol Reagent (Fisher Scientific). First strand cDNA was synthesized from 1 μg of total RNA using the Verso cDNA Synthesis Kit (Fisher Scientific). qPCR was performed in duplicate wells using the LightCycler 480 system (Roche Applied Science) and SYBR Green 1 Master Mix (Roche Applied Science) as follows: 40 cycles of amplification with 10 s at 95°C, 20 s at 60°C and 20 s at 72°C. After amplification, the melting curve was assessed to ensure the presence of a single product. Pooled RNAs were used as a calibrator and Ct values of the target gene were normalized to the Ct values of the house-keeping gene GAPDH. The expression level of each transcript was determined using the 2-ΔΔCt method. Primer sequences are described in Table 1.

**Table 1:** Primers used.

Gene	Forward primer	Reverse primer	Product length
atrogin-1	CGACCTCAGCAGTTACTGCAA	TTTGCTATCAGCTCCAACAG	288
MuRF1	AAACAGGAGTGCTCCAGTCGG	CGCCACCAGCATGGAGATACA	222
FoxO1	TTTGCGCCACCAAACACCAGTT	TGGCTGCCATAGGTTGACATGA	311
FoxO3	ACGTGATGCTTCGCAATGATCCGA	ACTCAAGCCCATGTTGCTGACA	158
myostatin	GAGCATTGATGTGAAGACAGTG	GTTACCTTGACCTCTAAAAACGG	162
IGF-1	TGAGCTGGTGGATGCTGTTGAGTT	TGCACTCCCTCTACTTGCGTTCTT	271

### Statistical analysis

The percentage of variation between “with” or “without” treatment was compared to a reference value of 100 using a paired Student’s *t*-test. To examine within-group changes, one-way analysis of variance followed by a Tukey’s multiple comparison test was performed to compare values, or the percentage of variation to a reference value of 100. Statistical analyses were performed with R 3.1.1. Significance was at P≤0.05.

## Results

### Characteristics of the study groups

The main clinical and functional characteristics of the study groups are presented in Table 2. The COPD patients showed a moderate to severe clinical state based on the following: post-bronchodilator forced expiratory volume in 1 second (FEV1%pred.) lower than 50% predicted, moderate exercise limitation and muscle dysfunction as indicated by moderately reduced 6-minute walking distance (6MWD) and quadriceps muscle voluntary contraction (qMVC) values compared with predicted values, and a high BODE index [18].

**Table 2:** Characteristics of the study groups.

	Healthy subjects	COPD patients
n	8	12
Gender (M/F)	7/1	9/3
Age (yrs)	62 [57.0–67.5]	59.0 [54.0–62.8]
BMI (kg/m^2^**)**	25.9 [24.3–27.8]	21.6 [19.0–26.0]
FFMI (kg/m^2^**)**	19.4 [18.4–20.5]	17.1 [15.0–18.3]
FEV_1_ (% pred.)	102.0 [93.0–105.0]	32.0 [26.8–47.5]
FEV_1_/FVC (%)	75.0 [68.8–81.5]	39.0 [32.0–41.0]
6MWD (% pred.)	88.0 [83.5–97.0]	68.0 [55.5–71.5]
qMVC (kg)	27.7 [25.1–31.6]	17.6 [12.9–32.2]
BODE index	-	5.0 [4.3–8.5]

Definition of abbreviations: BMI = body mass index; FFMI = fat-free mass index; FEV_1_ = forced expiratory volume in 1 second; FVC = forced vital capacity; 6MWD = 6-minute walking distance; qMVC = quadriceps muscle voluntary contraction. The BODE index takes into account the body mass index, the airflow obstruction, the functional dyspnea, and the exercise capacity [18]. Values are presented as median [25^th^ percentile-75^th^ percentile].

### Oxidative stress is induced in H_2_O_2_-treated COPD myotubes

**Fig 1:**
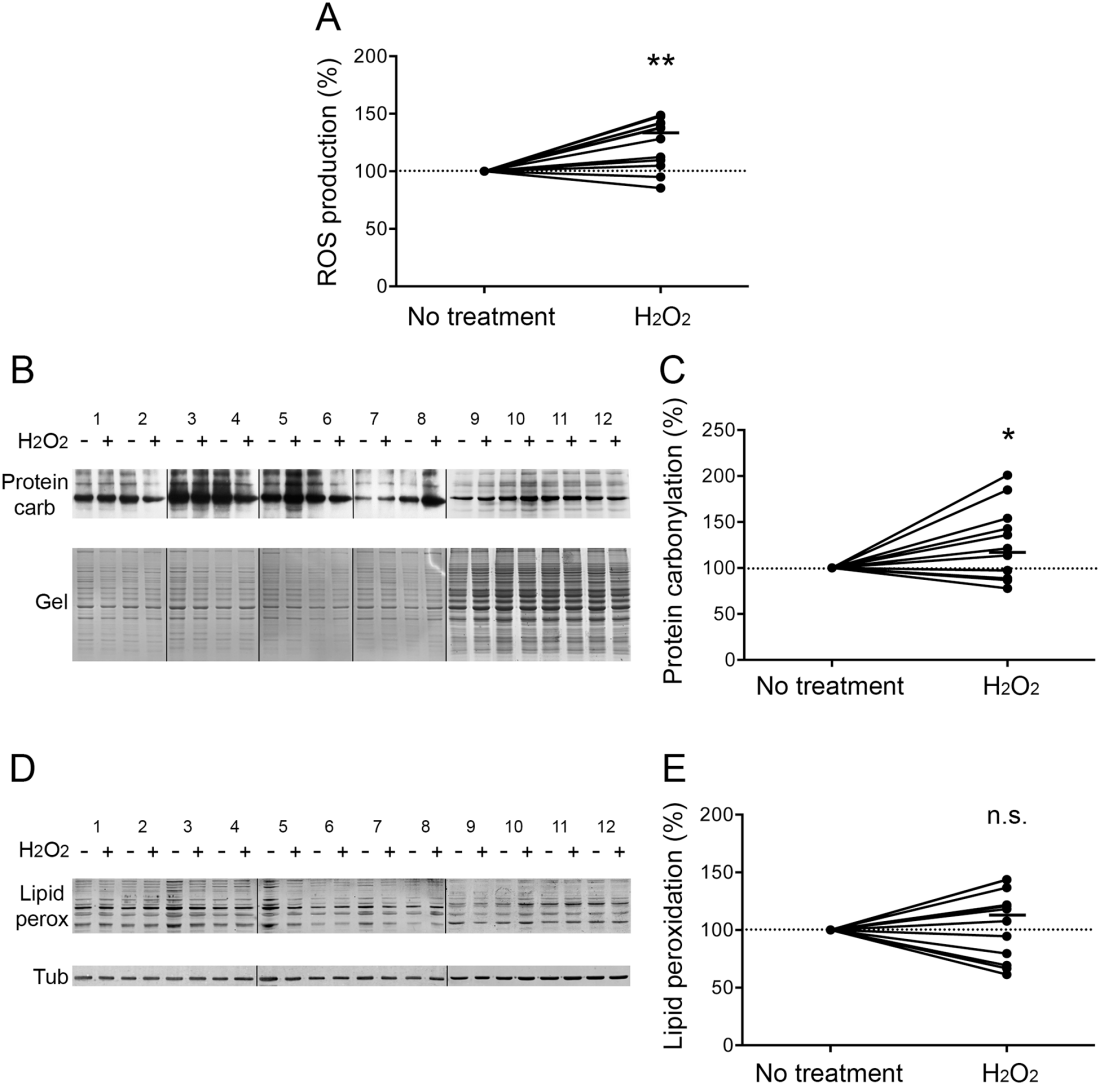
Oxidative stress status after H_2_O_2_ treatment. (A) The variation of ROS production in COPD myotubes is represented after H_2_O_2_ treatment. (B) Representative Western blots showing levels of protein carbonylation with or without H_2_O_2_ treatment, for the myotubes cultures derived from 12 COPD patients (1–12). Coomassie blue-stained gels are also presented for loading control. (C) Variation of protein carbonylation relative to Coomassie blue-stained bands after H_2_O_2_ treatment. (D) Representative Western blots showing levels of lipid peroxidation with or without H_2_O_2_ treatment in COPD cultured myotubes derived from 12 COPD patients (1–12). Tubulin detection is used as a loading control. (E) Variation of lipid peroxidation relative to tubulin expression levels following H_2_O_2_ treatment. (n.s.) indicates statistically non-significant. (*) and (**) indicate statistical significance at P≤0.05 and P<0.01, respectively. The medians are indicated.

Myotubes derived from COPD patients were treated with the pro-oxidant molecule H_2_O_2_. The production of reactive oxygen species (ROS) was significantly increased in these myotubes (P = 0.002; Fig 1A). Cellular damage was then assessed. The pro-oxidant treatment induced a significant increase in protein carbonylation (P = 0.050; Fig 1B and 1C), while no change was observed in the level of lipid peroxidation (P = 0.552; Fig 1D and 1E).

### Atrophy is induced in H_2_O_2_-treated COPD myotubes

**Fig 2:**
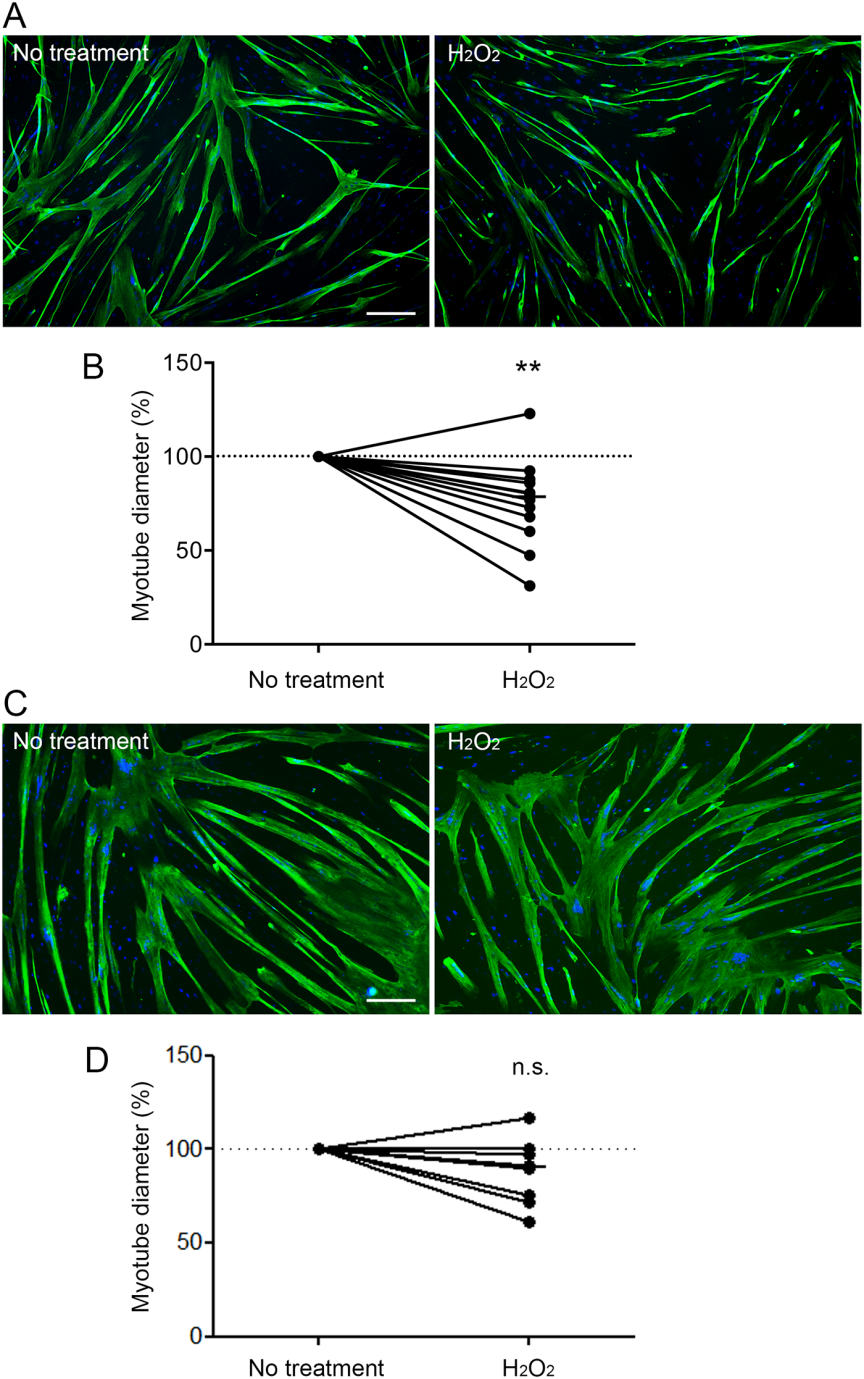
Diameter of COPD and healthy subject myotubes after H_2_O_2_ treatment. Representative images of COPD myotubes (A) or healthy subject myotubes (C) with or without H_2_O_2_ treatment, showing fluorescence double-labelling using an anti-troponin T antibody (green) and Hoechst (blue). Bar = 200 μm. Analysis of the variation of the myotube diameter after H_2_O_2_ treatment of the COPD myotubes (B) or the healthy subject myotubes (D). (**) indicates statistical significance at P<0.01. (n.s.) indicates statistically non-significant. The medians are indicated.

**Fig 3:**
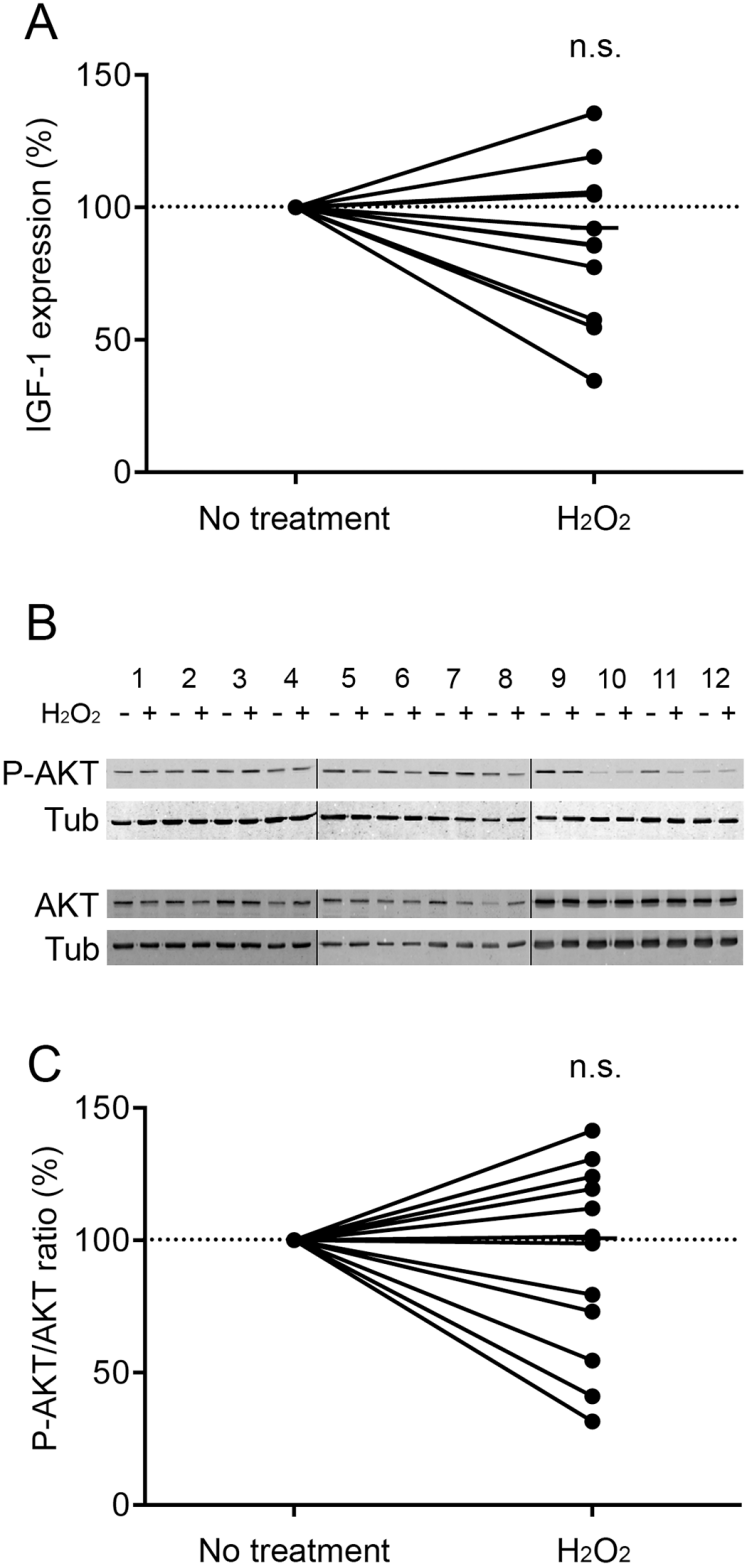
Expression levels of protein synthesis markers after H_2_O_2_ treatment. (A) Variation of IGF-1 mRNA expression in COPD myotubes after H_2_O_2_ treatment. (B) Representative Western blots showing expression levels of phosphorylated-AKT (P-AKT) and AKT in cultured COPD myotubes derived from 12 COPD patients (1–12), with or without H_2_O_2_ treatment. Tubulin expression is used as a loading control. (C) Variation of the P-AKT/AKT ratio relative to tubulin expression levels following H_2_O_2_ treatment. (n.s.) indicates statistically non-significant. The medians are indicated.

**Fig 4:**
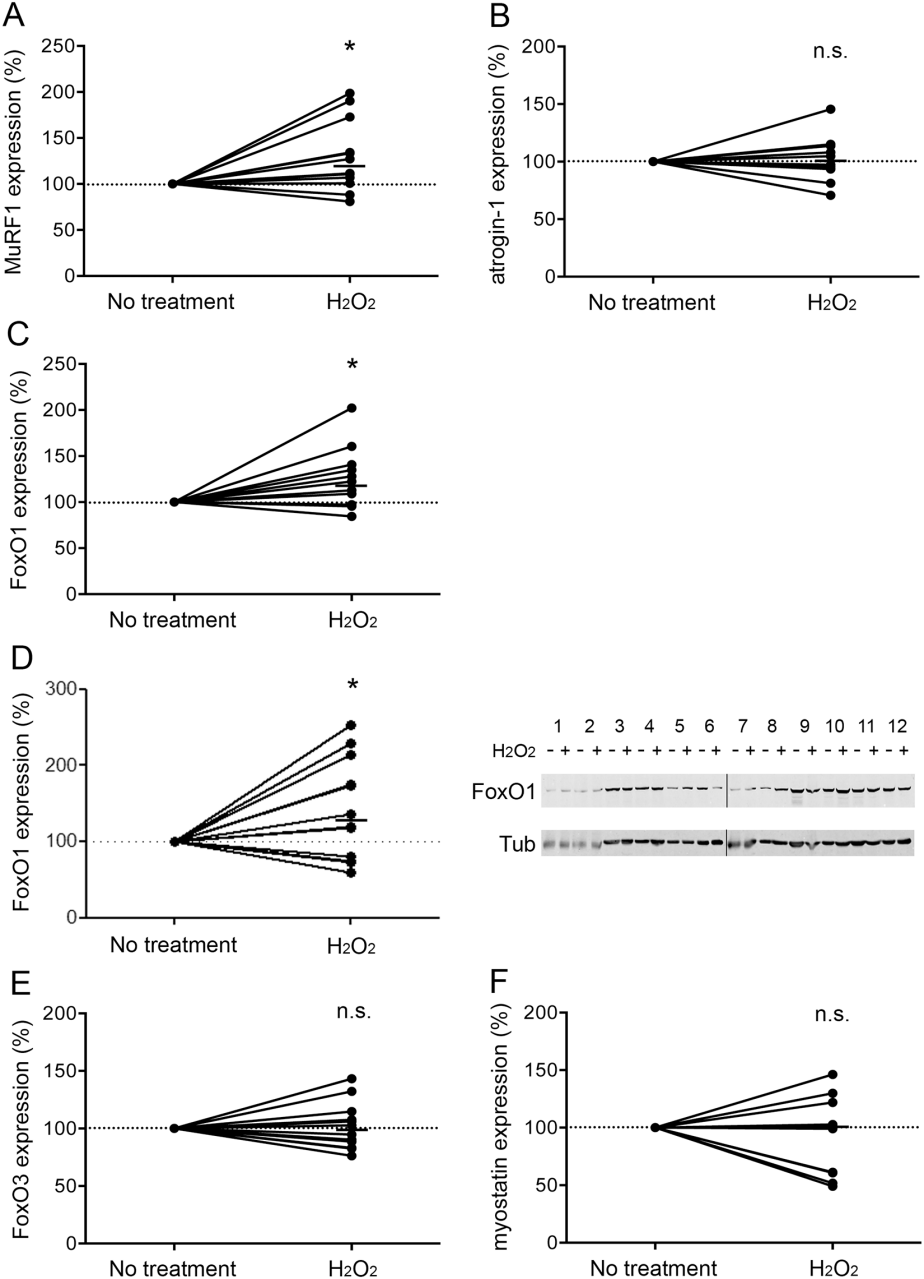
Expression levels of protein breakdown markers after H_2_O_2_ treatment. Variation of MuRF1 (A), atrogin-1 (B), FoxO1 (C), FoxO3 (E) and myostatin (F) RNA expression in COPD myotubes after H_2_O_2_ treatment. (D) Variation of FoxO1 protein expression relative to tubulin expression in COPD myotubes after H_2_O_2_ treatment, and representative Western blots showing expression levels of FoxO1 and tubulin in cultured COPD myotubes derived from 12 COPD patients (1–12), with or without H_2_O_2_ treatment. (n.s.) indicates statistically non-significant, and (*) indicates statistical significance at P≤0.05. The medians are indicated.

The diameter of the H_2_O_2_-treated COPD myotubes was then assessed by immunofluorescence microscopy. Representative images of treated and non-treated COPD myotubes derived from one COPD patient are shown in Fig 2A. Analysis of the cultured COPD myotubes showed a significantly reduced myotube diameter after H_2_O_2_ treatment (P = 0.003; Fig 2B). Interestingly, H_2_O_2_ treatment of myotubes derived from healthy subjects did not induce any significant reduction of the myotube diameter (P = 0.098; Fig 2C and 2D). We then assessed the expression levels of various markers of the protein synthesis and protein breakdown pathways in the H_2_O_2_-treated COPD myotubes. The RNA expression of IGF-1 and the P-AKT/AKT ratio, two markers of the protein synthesis pathway, were unchanged after pro-oxidant treatment (P = 0.185 and P = 0.475, respectively; Fig 3A–3C). Nevertheless, although the RNA expression of atrogin-1, FoxO3 and myostatin were not modified in the treated myotubes (P = 0.616, P = 0.762 and P = 0.510, respectively; Fig 4B, 4E and 4F), the MuRF1 and FoxO1 RNA expression levels were significantly more elevated in the H_2_O_2_-treated COPD myotubes (P = 0.022 and P = 0.030, respectively; Fig 4A and 4C). The increase of FoxO1 RNA expression levels in H_2_O_2_-treated COPD myotubes was confirmed at the protein level (P = 0.048; Fig 4D).

### Oxidative stress is reduced in ascorbic acid-treated COPD myotubes

**Fig 5:**
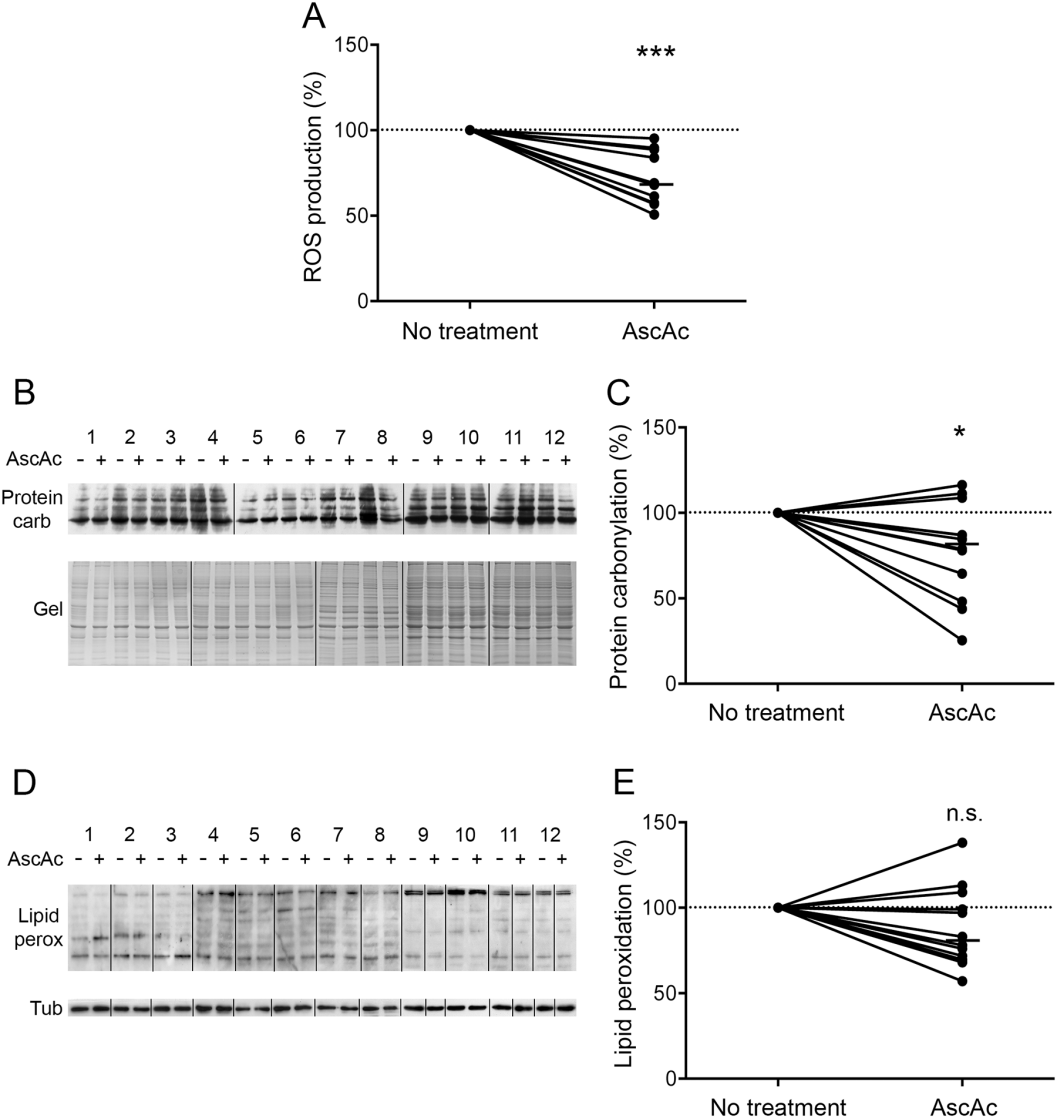
Oxidative stress status after ascorbic acid treatment. (A) The variation of ROS production in COPD myotubes is shown after ascorbic acid treatment. (B) Representative Western blots showing levels of protein carbonylation with or without ascorbic acid treatment for the myotubes cultures derived from 12 COPD patients (1–12). Coomassie blue-stained gels are used for loading control. (C) Variation of protein carbonylation relative to Coomassie blue-stained bands after ascorbic acid treatment. (D) Representative Western blots showing levels of lipid peroxidation with or without ascorbic acid treatment in COPD cultured myotubes derived from 12 COPD patients (1–12). Tubulin detection is used as a loading control. (E) Variation of lipid peroxidation relative to tubulin expression levels following ascorbic acid treatment. (n.s.) indicates statistically non-significant. (*) and (***) indicate statistical significance at P≤0.05 and P<0.001, respectively. The medians are indicated.

We previously showed that cultured COPD myotubes display elevated basal oxidative stress associated with atrophy compared with myotubes from healthy individuals [13]. In order to determine whether oxidative stress is involved in the atrophy of COPD myotubes *in vitro*, the myotubes were cultured in the presence of an antioxidant molecule, ascorbic acid. As shown in Fig 5A, ascorbic acid significantly reduced ROS production in the myotubes (P<0.001). Furthermore, although no change in lipid peroxidation was observed (P = 0.110; Fig 5D and 5E), protein carbonylation was significantly decreased in the ascorbic acid-treated COPD myotubes (P = 0.019; Fig 5B and 5C).

### Atrophy is decreased in ascorbic acid-treated COPD myotubes

**Fig 6:**
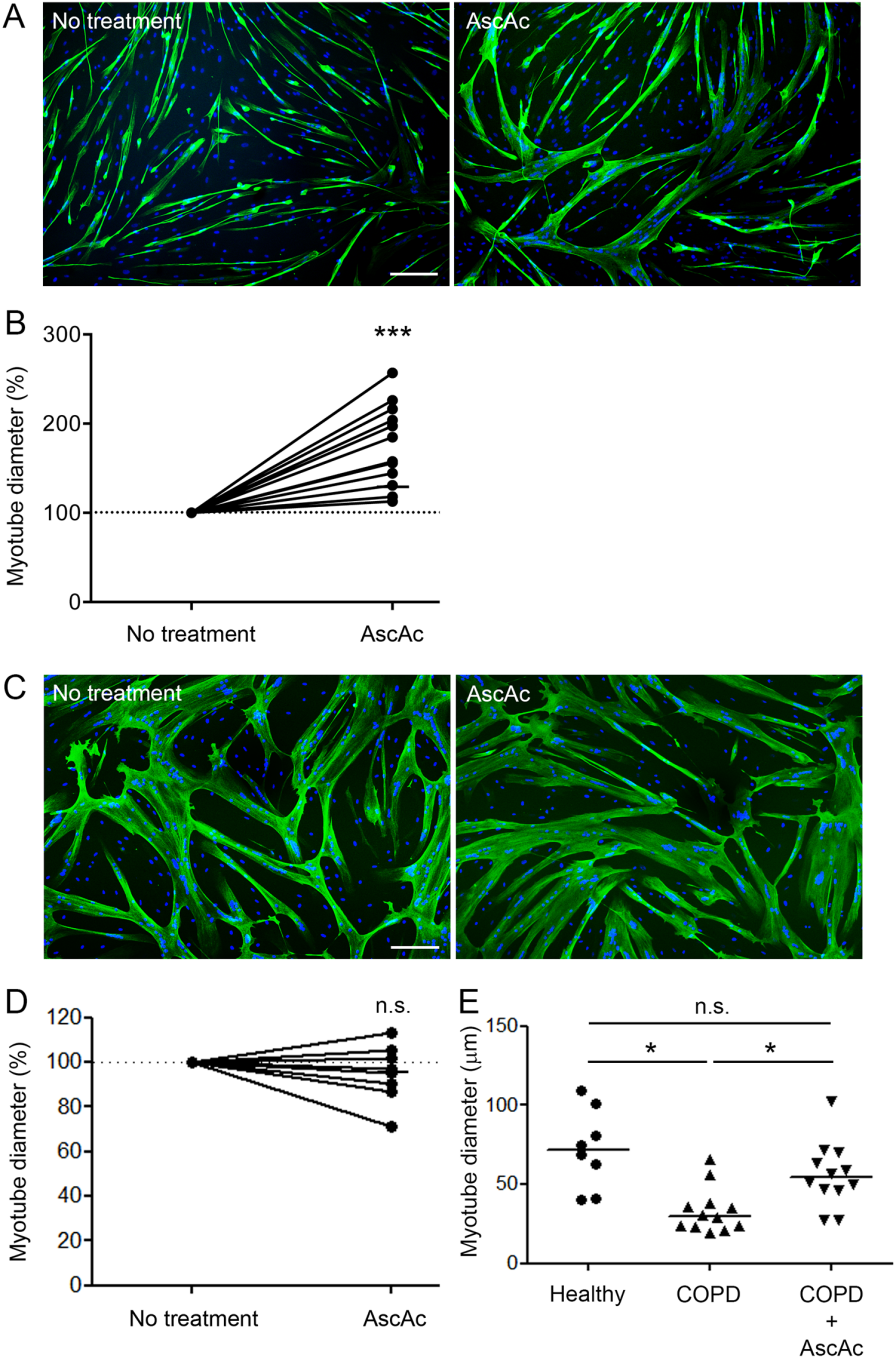
Diameter of COPD and healthy subject myotubes after ascorbic acid treatment. Representative images of COPD myotubes (A) or healthy subject myotubes (C) with or without ascorbic acid treatment, showing fluorescence double-labeling using an anti-troponin T antibody (green) and Hoechst (blue). Bar = 200 μm. Analysis of the variation of the COPD myotube diameter (B) or the healthy subject myotube diameter (D) after ascorbic acid treatment. (E) Diameter of myotubes from healthy subjects and COPD patients, cultured with or without ascorbic acid. (*) and (***) indicate statistical significance at P≤0.05 and P<0.001, respectively. (n.s.) indicates statistically non-significant. The medians are indicated.

**Fig 7:**
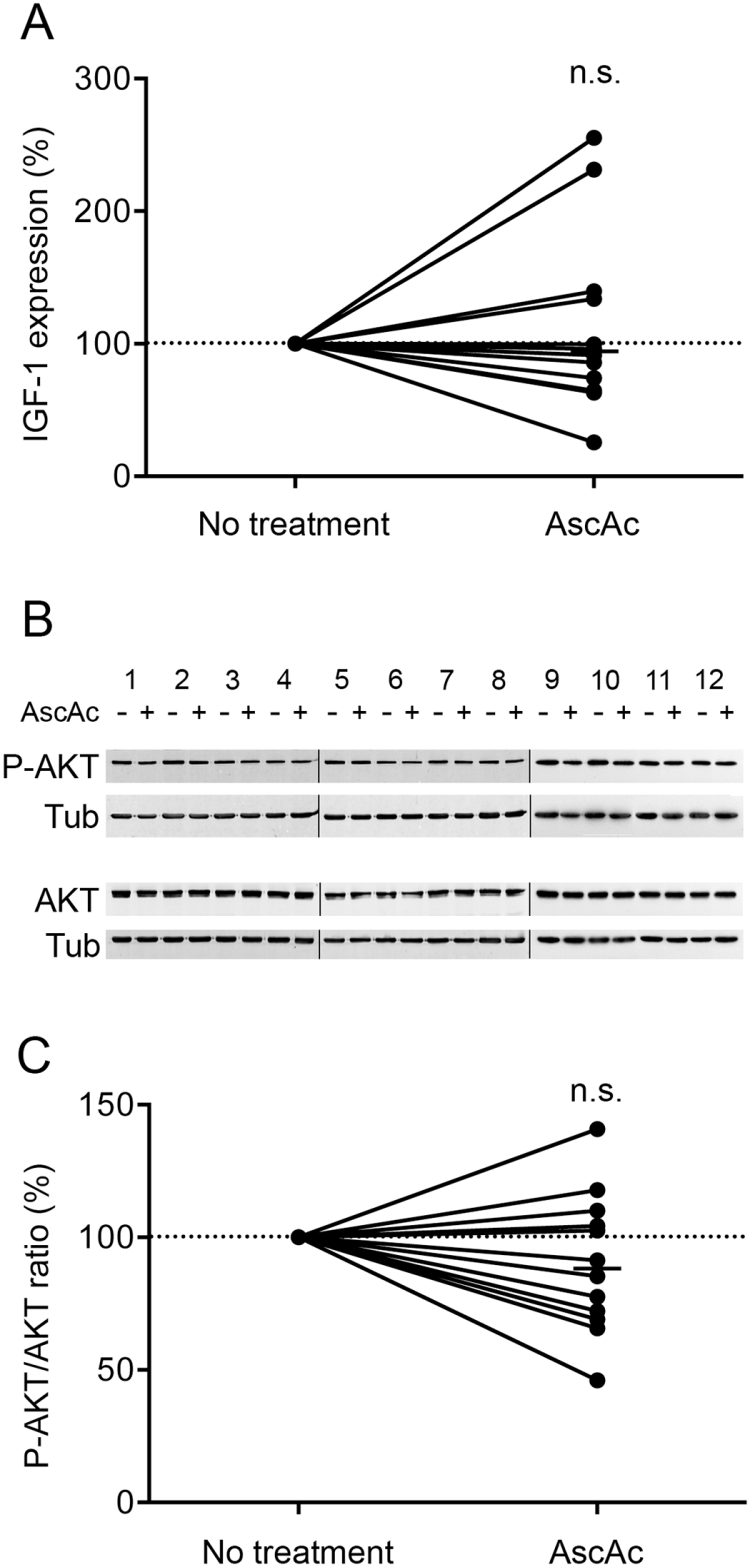
Expression levels of protein synthesis markers after ascorbic acid treatment. (A) Variation of IGF-1 mRNA expression in COPD myotubes after ascorbic acid treatment. (B) Representative Western blots showing expression levels of phosphorylated-AKT (P-AKT) and AKT in cultured COPD myotubes derived from 12 COPD patients (1–12), with or without ascorbic acid treatment. Tubulin expression is used as a loading control. (C) Variation of the P-AKT/AKT ratio relative to tubulin expression levels following ascorbic acid treatment. (n.s.) indicates statistically non-significant. The medians are indicated.

**Fig 8:**
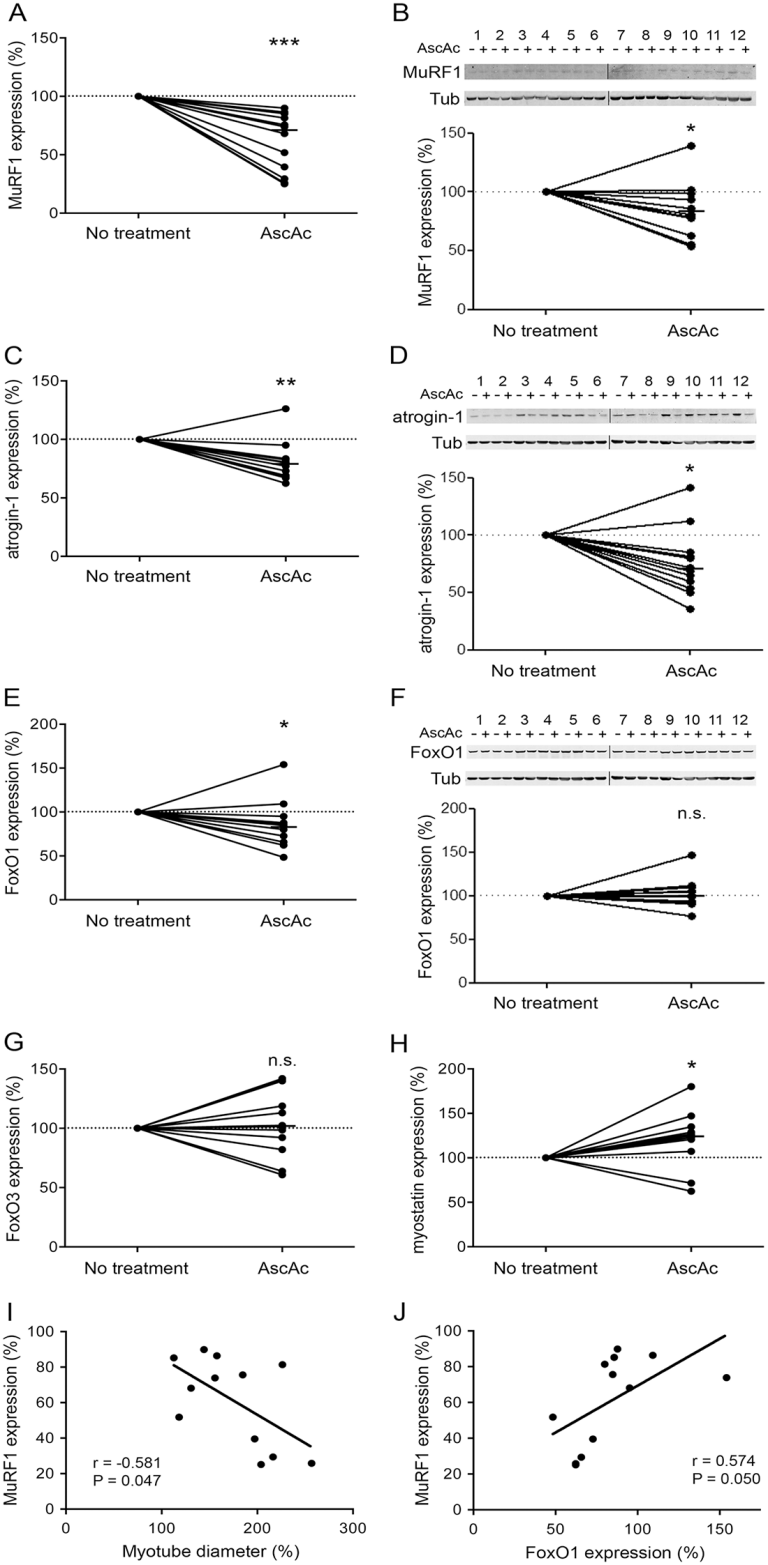
Expression levels of protein breakdown markers after ascorbic acid treatment. Variation of MuRF1 (A), atrogin-1 (C), FoxO1 (E), FoxO3 (G) and myostatin (H) RNA expression levels in COPD myotubes after ascorbic acid treatment. Variations of MuRF1 (B), atrogin-1 (D) and FoxO1 (F) protein expression relative to tubulin expression in COPD myotubes after ascorbic acid treatment, and the representative Western blots showing their expression levels as well as tubulin expression in cultured COPD myotubes derived from 12 COPD patients (1–12). (n.s.) indicates statistically non-significant. (*), (**) and (***) indicate statistical significance at P≤0.05, P<0.01 and P<0.001, respectively. The medians are indicated. Statistical analysis showing correlations between the variations in the RNA expression of MuRF1 and: (I) the variations in myotube diameter, and (J) the variations in the RNA expression of FoxO1, after ascorbic acid treatment.

The diameter of the treated COPD myotubes was then measured for each culture. Fig 6A shows representative pictures of COPD myotubes from one patient cultured in absence and in presence of ascorbic acid. Analysis of the cultures from the 12 COPD patients indicated a significant increase in the myotube diameter after treatment (P<0.001; Fig 6B). However, analysis of the ascorbic acid-treated myotubes derived from 8 healthy subjects showed no variation in the myotube diameter (P = 0.318; Fig 6C and 6D). It can be noted that treatment of the COPD myotubes with ascorbic acid was able to increase their diameter to a level similar to the diameter of healthy subject myotubes (Fig 6E). Expression of markers of the protein synthesis and protein breakdown pathways was then compared between ascorbic acid-treated and non-treated COPD myotubes. Both IGF-1 RNA expression and the P-AKT/AKT ratio showed no significant variation after antioxidant treatment (P = 0.510 and P = 0.222, respectively; Fig 7A–7C). Furthermore, no change was observed in FoxO3 RNA expression, while myostatin RNA expression was significantly increased following acid ascorbic treatment (P = 0.570 and P = 0.037, respectively; Fig 8G and 8H). This treatment nevertheless induced a significant decrease in the RNA expression levels of MuRF1, atrogin-1 and FoxO1 (P<0.001, P = 0.002 and P = 0.042, respectively; Fig 8A, 8C and 8E). These decreased RNA expression levels in ascorbic acid-treated myotubes were confirmed at the protein level for MuRF1 and atrogin-1 (P = 0.049 and P = 0.012, respectively; Fig 8B and 8D), but not for FoxO1 (P = 0.741; Fig 8F). We also observed a significant negative correlation (r = -0.581; P = 0.047) between the variations in myotube diameter and the variations in the RNA expression of MuRF1 after ascorbic acid treatment (Fig 8I), and a significant positive correlation (r = 0.574; P = 0.050) between the variations in MuRF1 and FoxO1 expression under the same conditions (Fig 8J).

### H_2_O_2_-induced atrophy is reversed by ascorbic acid treatment

**Fig 9:**
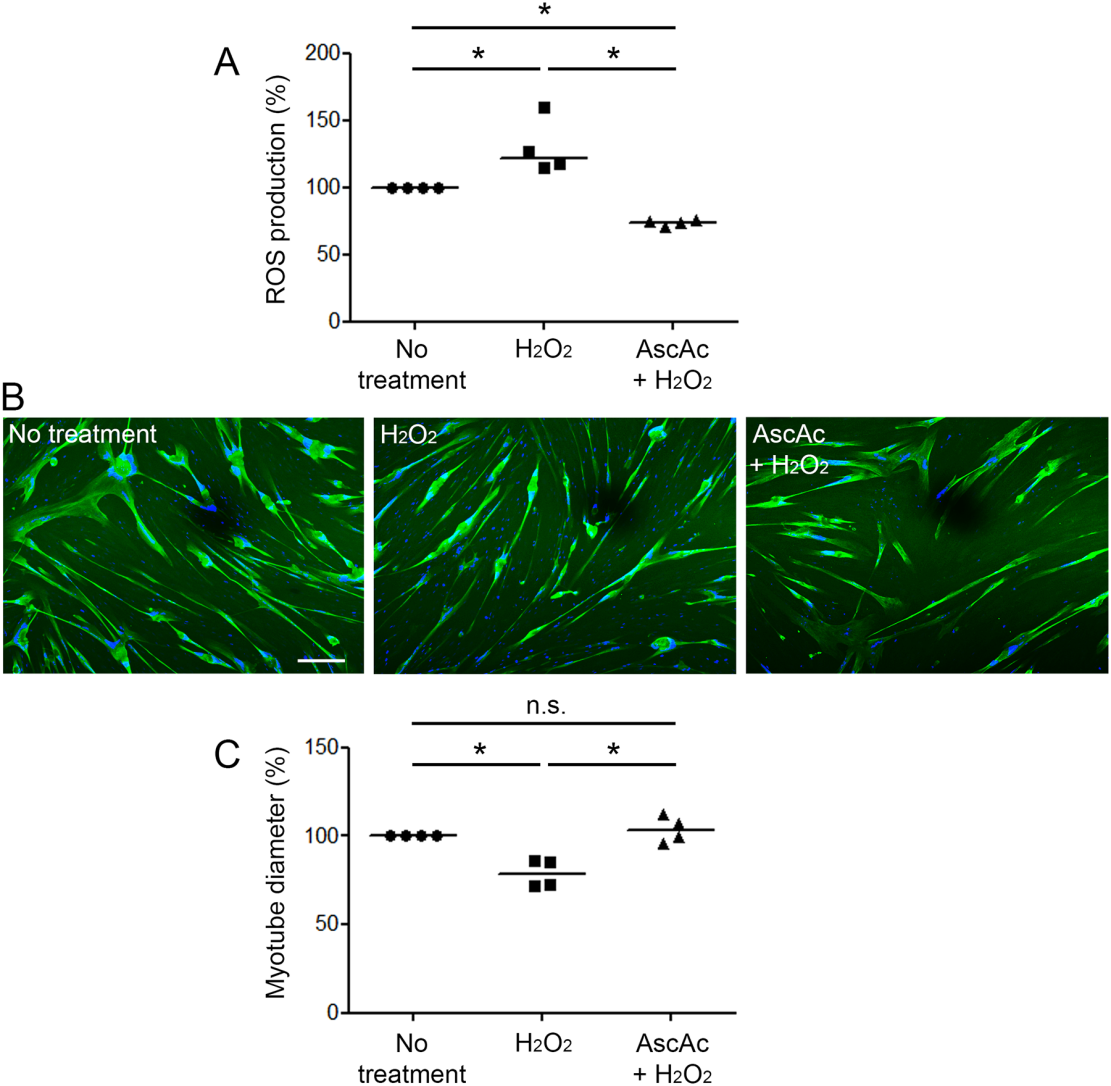
ROS production and COPD myotube diameter after both H_2_O_2_ and ascorbic acid treatment. (A) The variation of ROS production in myotubes derived from 4 COPD patients is shown after H_2_O_2_ treatment, and both H_2_O_2_ and ascorbic acid treatment. (B) Representative images of COPD myotubes from one patient, in absence of treatment, after H_2_O_2_ treatment or after both H_2_O_2_ and ascorbic acid treatment, showing fluorescence double-labeling using an anti-troponin T antibody (green) and Hoechst (blue). Bar = 200 μm. (C) Analysis of the variation of the myotube diameter to a reference value of 100after H_2_O_2_, and both H_2_O_2_ and ascorbic acid treatments. (n.s.) indicates statistically non-significant. (*) indicates statistical significance at P≤0.05. The medians are indicated.

We next determined the effect of an ascorbic acid treatment on the H_2_O_2_-induced atrophy of cultured COPD myotubes. Ascorbic acid significantly reduced the H_2_O_2_-induced ROS increase observed in COPD myotubes (P≤0.05; Fig 9A). Representative pictures of myotubes derived from one COPD patient in absence of treatment, in presence of H_2_O_2_, and in presence of both ascorbic acid and H_2_O_2_ are shown in Fig 9B. Analysis of the COPD myotube diameter indicated that the decreased myotube diameter observed in H_2_O_2_-treated myotubes was fully reverted by ascorbic acid (P≤0.05; Fig 9C).

### Atrophy is reduced in MG132-treated COPD myotubes

**Fig 10:**
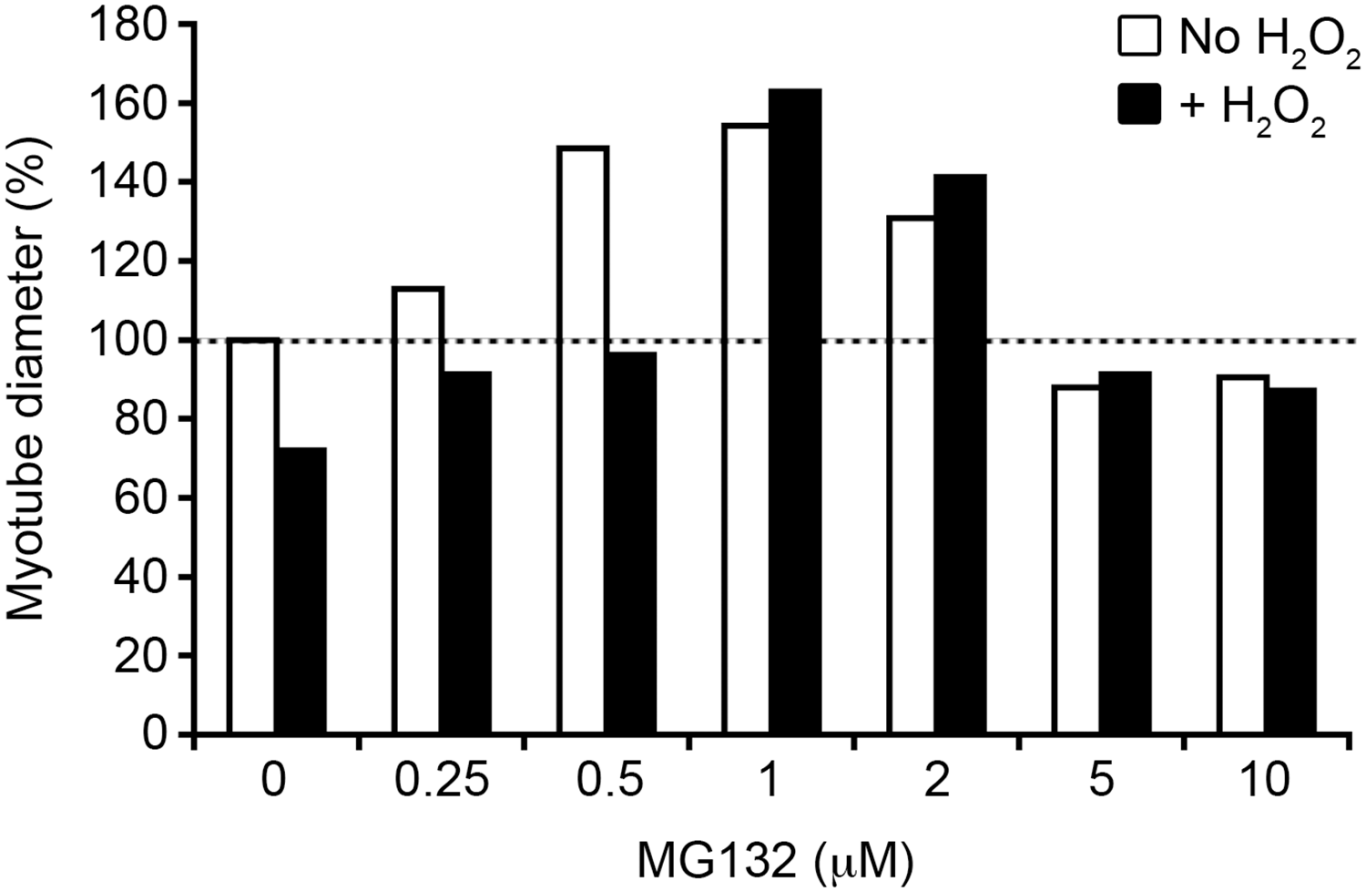
COPD myotube diameter after MG132 treatment. Analysis of the variation of the diameter of COPD myotubes derived from one patient after treatment with increasing concentrations of the proteasome inhibitor MG132, in absence (white bars) or in presence (black bars) of the pro-oxidant molecule H_2_O_2_. Results are from one of two separate experiments that yielded similar results. The mean diameter of COPD myotubes cultured in absence of MG132 and H_2_O_2_ is considered as the reference value (100%).

To further investigate the involvement of the ubiquitin/proteasome pathway in the oxidative stress-induced atrophy of COPD myotubes, increasing concentrations of the proteasome inhibitor MG132 were added to the culture medium. MG132 treatment reduced the atrophy of the COPD myotubes, with a maximal effect reached at 1 μM MG132 (Fig 10, white bars). Interestingly, in presence of the pro-oxidant molecule H_2_O_2_, low concentrations of MG132 (up to 0.5 μM) prevented the H_2_O_2_-induced atrophy of the COPD myotubes, while higher concentrations (1 to 2 μM) increased the diameter of the myotubes to a level similar to that observed in absence of H_2_O_2_ (Fig 10, black bars).

## Discussion

Although oxidative stress has been considered as one of the mechanisms that leads to muscle wasting in COPD patients, its involvement has never been demonstrated. In the present study, we showed that pro-oxidant treatment of cultured COPD myotubes induced a rise in cellular ROS production associated with greater cellular damage (Fig 1). Moreover, the increased oxidative stress level was associated with a more pronounced atrophy of the COPD myotubes, as reflected by a reduced diameter (Fig 2), and activated atrophic signaling (Fig 4). Most interestingly, an antioxidant treatment was used to reduce the elevated basal oxidative stress level observed in cultured COPD myotubes [13], which resulted in a decrease in ROS production and the oxidative stress-induced cellular damage (Fig 5), in association with an increase in COPD myotube diameter to a level similar to the diameter of healthy subject myotubes (Fig 6) and a decrease in atrophic signaling (Fig 8). Taken together, our findings showed that the ubiquitin/proteasome system, *via* the FoxO1/MuRF1/atrogin-1 signaling pathway, is involved in the oxidative stress-induced atrophy of COPD myotubes *in vitro*.

Satellite cells purified from skeletal muscle biopsies are increasingly used to study the signaling pathways involved in the muscle dysfunction of inherited and chronic pathologies such as facioscapulohumeral muscular dystrophy (FSHD), diabetes and COPD. For example, myoblast susceptibility to oxidative stress and the morphological appearance of myoblasts and myotubes have been assessed in cells derived from FSHD satellite cells [19,20]. Furthermore, the inflammation-dependent molecular mechanisms leading to the maintenance of the insulin response have been compared in satellite cell-derived myotubes from insulin-resistant and insulin-sensitive obese subjects [21]. Satellite cells from the muscle biopsies of COPD and healthy subjects have also been used to study muscle regeneration and atrophy in COPD patients [13,22]. These cultured satellite cells therefore appear to be useful *in vitro* cellular models to study the cellular mechanisms that are affected in pathological muscles.

We previously showed that cultured COPD myotubes displayed elevated basal oxidative stress associated with atrophy and were also more susceptible to an H_2_O_2_-induced oxidative stress compared with myotubes from healthy individuals [13]. Furthermore, with the aim of studying atrophic signaling in muscle, it was demonstrated that treating the myogenic C2C12 cell line with H_2_O_2_ stimulated protein catabolism by activating the ubiquitin/proteasome system [11] and thus directly induced muscle atrophy [12]. In accordance with previous studies [12,13], we therefore subjected COPD myotubes to pro-oxidant treatment by brief exposure to H_2_O_2_ at a concentration level below the threshold of the deleterious effects leading to cell death. At this low concentration, which potentially mimics such physiological conditions as increased ROS production during exercise, we demonstrated that H_2_O_2_-induced oxidative stress promoted increased ROS production associated with cellular damage, leading to enhanced atrophy, while protein synthesis signaling was not affected. Interestingly, the *in vitro* COPD myotubes subjected to this H_2_O_2_-induced oxidative stress exhibited characteristics of elevated oxidative stress and atrophy similar to those of *in vivo* peripheral muscles from COPD patients after physical activity. Indeed, exercise has been found to induce systemic and muscle oxidation of the proteins and lipids associated with muscle wasting in COPD patients [7,9], but this causal relation was only supported by correlations. On the contrary, we have shown that treatment of healthy subject myotubes with H_2_O_2_ did not induce any reduction of the myotube diameter, suggesting that cultured healthy myotubes are better able to counteract oxidative stress than COPD myotubes.

To lower the elevated basal oxidative stress in the *in vitro* myotubes derived from COPD patients, we treated these cultured myotubes with the antioxidant molecule, ascorbic acid. Some *in vitro* studies have assessed the effects of antioxidant and/or anti-inflammatory molecules on the atrophic signaling pathways of muscle cells. Isoflavones have been shown to reverse C2C12 muscle cell atrophy caused by TNF-α through the inhibition of MurF1 expression [23,24], while resveratrol treatment of dexamethasone-atrophied L6 myotubes prevented muscle atrophy in association with repression of atrogin-1 and MuRF1 expression [25]. Although the oxidative stress status of the muscle cells was not assessed in these studies, the findings suggested that the increased atrophic signaling in the cultured myotubes could be prevented by antioxidant treatment. However, only a few clinical trials with COPD patients have studied the impact of antioxidant supplementation on the function of peripheral muscles. A pressurized whey supplementation potentiated the effects of exercise training but with no change in the assessed oxidative stress markers [26], while intravenous ascorbate administration attenuated quadriceps fatigue in association with decreased oxidative stress levels [27]. More interestingly, N-acetylcysteine treatment prevented exercise-induced oxidative stress damage and improved quadriceps endurance in COPD patients [8]. The present study showed that ascorbic acid treatment can lower ROS production, oxidation-induced cellular damage, and atrophy in cultured COPD muscle cells, and it is therefore one of the first demonstrations of the beneficial impact of antioxidant treatment on the altered COPD peripheral muscles. It can be noted that the ascorbic acid treatment had no effect on the diameter of healthy subject myotubes in culture but was able to increase the diameter of COPD myotubes to a level similar to the diameter of healthy subject myotubes, strengthening its potential favorable effect on atrophied muscle cells. Furthermore, we have shown that ascorbic acid is also able to prevent the H_2_O_2_-induced atrophy of COPD myotubes, highlighting the involvement of oxidative stress in the atrophy of COPD muscle cells. Interestingly, the increase in the COPD myotube diameter after ascorbic acid treatment was correlated with the decreased expression of the atrophic marker MurF1, while the FoxO1 and MuRF1 expression decreases were positively correlated, suggesting that the ubiquitin/proteasome system is indeed involved in the oxidative stress-induced atrophy of COPD myotubes *in vitro*.

It has been amply documented that skeletal muscle atrophy involves the increased expression of two E3 ubiquitin ligases, MuRF1 and atrogin-1, under the control of the FoxO family of transcription factors, and in particular FoxO1, leading to the activation of protein degradation *via* the ubiquitin/proteasome system [28–30]. Furthermore, ROS-mediated activation of FoxO was observed in response to skeletal muscle atrophy during hindlimb immobilization [31]. Here, we showed that ascorbic acid treatment of cultured COPD myotubes reduced myotube atrophy and decreased the expression of FoxO1, MuRF1 and atrogin-1, whereas the pro-oxidant molecule H_2_O_2_ led to increased atrophy with upregulation of MuRF1 and FoxO1 expression. Interestingly, treatment of the myotubes with the proteasome inhibitor MG132, which downregulates MuRF1 and atrogin-1 expression in skeletal muscle cells [32], restored the basal atrophy level of the COPD myotubes to a level similar to that observed with ascorbic acid. Moreover, at higher concentrations, MG132 was able to totally suppress the H_2_O_2_-induced COPD myotube atrophy. Recently, the MG132-induced downregulation of MuRF1 and atrogin-1 expression in an *in vivo* mouse model of skeletal muscle wasting prevented immobilization-induced muscle atrophy and accelerated the rate of rehabilitation following hindlimb immobilization [32]. These results thus suggest that proteasome inhibitors are promising pharmacological tools to study the involvement of the ubiquitin/proteasome system in the skeletal muscle atrophy in COPD patients.

In summary, this study shows that the antioxidant treatment of cultured COPD myotubes leads to reduced ROS production, cellular damage, ubiquitin/proteasome signaling and atrophy, while pro-oxidant treatment induces the opposite effects. These data are therefore the first to show the involvement of oxidative stress in the atrophy of COPD peripheral muscle cells *in vitro*, *via* the FoxO1/MuRF1/atrogin-1 signaling pathway of the ubiquitin/proteasome system. Nevertheless, this mechanism does not exclude the participation of other cellular pathways in muscle mass homeostasis, pathways that will be assessed using the *in vitro* cellular model used in the present study.
